# Malignant paranasal sinus schwannoma

**DOI:** 10.1590/S1808-86942012000400026

**Published:** 2015-10-20

**Authors:** Sharlene Castanheira Pádua, Vítor Yamashiro Rocha Soares, André Luís de Queiroz, Márcio Nakanishi, Luiz Augusto Nascimento

**Affiliations:** 1 Graduation (Otorhinolaryngologist, Brasília University); 2 Graduation (Otorhinolaryngology Medical Resident, Brasília University); 3 Graduation (Assistant Professor, Department of Otorhinolaryngology and Head and Neck Surgery, Brasília University); 4 PhD in Sciences, ENT Department, University of São Paulo (Adjunct Professor, Department of Otorhinolaryngology and Head and Neck Surgery, Brasília University); 5 PhD in Surgery, ENT Department, Federal University of São Paulo (Adjunct Professor, Department of Otorhinolaryngology and Head and Neck Surgery, Brasília University)

**Keywords:** epistaxis, nasal obstruction, paranasal sinus neoplasms

## INTRODUCTION

Malignant schwannomas are tumors that arise from the Schwann cells of the peripheral nerves^1^. They represent 10% of all soft-tissue sarcomas, and are rare in the head and neck region. The extremities, trunk and thorax are the locations most commonly affected. When their origin is the paranasal sinuses, the trigeminal nerve is usually involved^2^. The interest in these tumors is their rarity and nonspecific symptoms in the nasal cavity. This means that they remain unsuspected and are often diagnosed late^2^, ^3^.

## CASE REPORT

**Figure 1:**
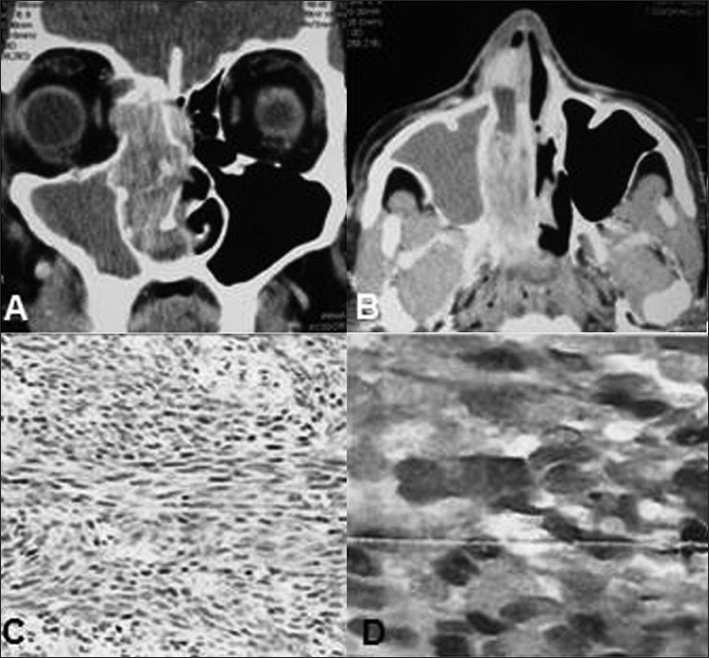
Contrast-enhanced axial (A) and coronal (B) computed tomography of the paranasal sinus, showing a voluminous mass in the right nasal cavity that extended as far as the pterygopalatine fossa, with erosion of the medial wall of the maxillary sinus, lamina papyracea and anterior and posterior ethmoid cells. Histopathological examination revealed a malignant neoplasm represented by atypical fusiform cells in fascicular arrangement, with great alternation of cellularity (HE, 40x) (C). Immunohistochemical analysis showed positivity for the S-100 protein (D).

A 57-year-old smoker and heavy drinker, who was a truck driver, came to the Otorhinolaryngology and Head and Neck Surgery Service of Brasília University Hospital presenting nasal obstruction, rhinorrhea and epistaxis of three years duration in the right nasal cavity (RNC), along with ipsilateral facial pain. Two months earlier, he noticed epiphora and exophthalmia in his right eye with unaffected visual acuity, but with hearing loss in his right ear. Physical examination showed a bent nose, with left axis deviation of the nasal septum, and a friable mass inside the RNC, showing hemorrhagic spots. Computed tomography (CT) on the paranasal sinus revealed a voluminous mass in the RNC, extending as far as the pterygopalatine fossa, measuring approximately 60 × 70 × 20 mm and eroding the medial wall of the maxillary sinus, the lamina papyracea, the anterior and posterior ethmoid cells and part of the wing of the sphenoid bone (Figure 1A-B).

Endoscopic resection of the mass was attempted and a friable brownish lesion with its epicenter probably in the posterior ethmoid was seen, along with infiltration of adjacent tissue. A biopsy was performed and the histopathological examination revealed a malignant neoplasia represented by atypical fusiform cells in a fascicular arrangement, with great alternation of cellularity and areas with intense collagen deposition, and with frequent mitotic figures and focal areas of necrosis. Immunohistochemical analysis showed positivity for the S-100 protein and type IV collagen (Figure 1C-D). The histological and immunohistochemical findings were compatible with malignant tumors of the peripheral nerve sheath.

The patient then underwent another procedure to check on the surgical margin status, using an open approach via lateral rhinotomy. The orbit was preserved. There wasn't tumor infiltration beyond the margin of resection. Postoperative radiotherapy was administered to reduce the risk of the disease recurrence. Up to the present moment (24 months after the operation), the patient has not presented any signs of tumor recurrence or distant metastases.

## DISCUSSION

Malignant schwannomas are sarcomas that arise from Schwann cells of the peripheral nerves. They represent approximately 10% of soft-tissue sarcomas, and are unusual in the head and neck region (8% of the cases)^1^, ^2^. The most common locations are the extremities, trunk, chest and retroperitoneum^1^, ^2^. Several studies have shown that malignant schwannomas in the nasal cavity, paranasal sinus and nasopharynx are rare^3^, ^4^. In the latter region, the ethmoid and maxillary sinuses are most often affected, the nasal fossa and sphenoid sinus come next and the frontal sinus is rarely affected^5^. There are few cases of benign schwannomas in national literature^6^, just Pinto et al.^7^ cited an unique case of ethmoid malignant schwannoma in a study about nasal and paranasal sinuses malignant tumors.

Malignant schwannomas of the paranasal sinuses usually originate in the trigeminal nerve, usually from the ophthalmic or maxillary division and their terminal branches^2^. Individuals in their fourth and fifth decades are more often affected, and there is no preference for sex or race^1^. They can occur alone, but are associated with von Recklinghausens disease in 30% of the cases, and usually evolve from malignant transformation of neurofibroma^1^, ^2^, ^5^, ^8^.

Microscopically, malignant tumors of the peripheral nerve sheath are characterized by layers of fusiform cells, with indistinct outlines and a moderate amount of cytoplasm. The nuclei are ovoid or fusiform with cellular and atypical pleomorphism. The mitotic activity level is variable and indicates the aggressiveness of the tumor^1^, ^3^, ^8^. Immunohistochemical analysis is positive for the S-100 protein. The differential diagnosis is made with fibrosarcoma, malignant fibrous histiocytoma, benign schwannoma, capillary hemangioma, hemangiopericytoma and other tumors^2^, ^3^, ^8^.

The standard treatment consists of resection of the tumor with safety margins. Lymph node disease occurs infrequently. Distant metastases may involve the lungs, and are associated with intracranial invasion^3^, ^5^. The role of radiotherapy and chemotherapy remains controversial. However, some authors have indicated radiotherapy in cases in which the tumor cannot be completely resected^2^. In the present, we indicated radiotherapy to reduce the risk of disease recurrence because it was performed piece meal endoscopic resection and histopathological study evidenced infiltration of adjacent tissue. The five-year survival rate is 65.7% among individuals presenting malignant schwannoma alone, and falls by 30% when associated with type 1 neurofibromatosis. Other prognostic factors include the degree of cell pleomorphism, level of mitotic activity and size of the primary tumor^2^, ^3^, ^8^. The present case did not present type 1 and presented an excellent result from surgery combined with radiation therapy. Currently, the patient does not present any signs of tumor recurrence or distant metastases.

## FINAL COMMENTS

Malignant tumors of the peripheral nerves sheath are rare in the head and neck region. The definitive diagnosis is only given through histological and immunohistochemical evaluations, with positive findings of S-100 positive. The standard treatment consists of complete resection of the tumor, with safety margins.
